# Anlotinib Suppressed Ovarian Cancer Progression via Inducing G2/M Phase Arrest and Apoptosis

**DOI:** 10.3390/jcm12010162

**Published:** 2022-12-25

**Authors:** Yanghui Zhu, Xiaoyu Wang, Zhaoyang Chen, Lingyan Zhou, Xiangjie Di, Ping Fan, Zhiyao He

**Affiliations:** 1 Department of Pharmacy, State Key Laboratory of Biotherapy and Cancer Center, West China Hospital, Sichuan University, Chengdu 610041, China; 2 Clinical Trial Center/NMPA Key Laboratory for Clinical Research and Evaluation of Innovative Drug, West China Hospital, Sichuan University, Chengdu 610041, China; 3 Key Laboratory of Drug-Targeting and Drug Delivery System of the Education Ministry, West China School of Pharmacy, Sichuan University, Chengdu 610041, China

**Keywords:** ovarian cancer, anlotinib, cell proliferation, angiogenesis, apoptosis

## Abstract

Ovarian cancer remains the most common gynecologic malignancy, because of its chemotherapy resistance and relapse. Anlotinib, a new oral multi-targeted tyrosine kinase inhibitor, has shown encouraging antitumor activity in several preclinical and clinical trials, while its effect on ovarian cancer has not been reported. In this study, we investigated the antitumor activity and underlying mechanism of anlotinib in ovarian cancer. Cell viability was analyzed by Cell Counting Kit-8 assay. Migration was measured by wound-healing assay. The cell cycle distribution and cell apoptosis rate were detected by flow cytometry. In vivo antitumor effect was analyzed in mouse ovarian carcinoma peritoneal metastasis model. We found that anlotinib inhibited the proliferation of ovarian cancer cells in a dose- and time- dependent manner by inducing G2/M phase arrest and apoptosis. Moreover, anlotinib upregulated the the phosphorylation of Histone H3, and expression of p21 protein in vitro. In addition, anlotinib inhibited the migration of ovarian cancer cells in vitro. Furthermore, anlotinib inhibited tumor growth by inhibiting cell proliferation and suppressing ovarian cancer angiogenesis in vivo. This study demonstrated the extraordinary anti-ovarian cancer effect of anlotinib, which may provide a promising therapeutic strategy for ovarian cancer.

## 1. Introduction

Ovarian cancer is one of the most common gynecologic malignancies. Worldwide, nearly 313,000 new cases of ovarian cancer were diagnosed, and 207,252 deaths occurred in 2020 [1]. Epithelial ovarian cancer (EOC) is the most common ovarian cancer, accounting for 90% [2]. Since no effective screening strategies are available, ovarian cancer is usually diagnosed at an advanced stage, resulting in a low overall survival rate [3,4]. In the status quo, the mainstay of treatment in ovarian cancer is cytoreductive surgery to R0, followed by adjuvant chemotherapy [5]. The-first line chemotherapy for EOC is carboplatin with paclitaxel [5], and targeted therapy includes angiogenesis inhibitors and poly adenosine diphosphate ribose polymerase inhibitors [6]. While >80% of patients initially respond to therapy, the majority ultimately recur and eventually develop chemotherapy-resistant disease [7].

Anlotinib is a novel oral multitarget tyrosine kinase inhibitor, which has been approved by National Medical Products Administration of China as a third-line treatment option for patients with advanced non-small cell lung cancer in 2018 [8,9]. Anlotinib mainly targets vascular endothelial growth factor receptor 2 (VEGFR2) [10], platelet-derived growth factor receptors β, receptor tyrosine kinase and fibroblast growth factor receptor (FGFR) [11,12,13], which has shown excellent antitumor effects on various solid tumors [14,15], such as hepatocellular carcinoma, metastatic soft-tissue sarcoma [12,16], pancreatic cancer [17], intrahepatic cholangiocarcinoma [18], colorectal carcinoma [19,20], gastric cancer [21] and thyroid cancer [22,23]. Although, several clinical trials of anlotinib in treating ovarian cancer are in progressing, and its clinical efficacy and underlying molecular mechanisms remain poorly understood.

In this study, we determined the antitumor effects of anlotinib on ovarian cancer cells. We demonstrated that anlotinib inhibited ovarian cancer cells proliferation by G2/M phase arrest and upregulated p21 protein expression, and promoted ovarian cancer cells apoptosis both in vivo and in vitro. Anlotinib inhibited ovarian cancer cells migration in vitro. In addition, we found that anlotinib inhibited angiogenesis in ovarian carcinoma peritoneal metastasis model. Thus, we identified that anlotinib could effectively inhibit ovarian cancer cells. This study provided promising evidence for the clinical therapeutic application of anlotinib in patients with ovarian cancer.

## 2. Materials and Methods

### 2.1. Cell Line and Culture

The human ovarian cancer cell line SKOV-3 (ATCC ^®^ HTB-77 ^TM^, RRID:CVCL_0532) was obtained from American Type Culture Collection. SKOV-3 cells were grown in McCoy’s 5A medium (Gibco, Grand Island, NY, USA) containing 10% fetal bovine serum (Gibco, Thornton, NSW, Australia) and penicillin-streptomycin (penicillin 100 U/mL, streptomycin 100 mg/mL, HyClone, Logan, UT, USA) in a 37 °C humidified atmosphere with 5% CO_2_.

### 2.2. Antibodies and Reagents

Antibodies against histone H3 (1:1000) (Cell Signaling Technology Cat# 9715), phospho-histone H3 (Ser10) (1:1000) (Cell Signaling Technology Cat# 0701), phospho-histone H2A.X (Ser139) (1:1000) (Cell Signaling Technology Cat# 9718), p21 (1:1000) (Cell Signaling Technology Cat# 2947, RRID:AB_823586), GAPDH (1:1000) (Cell Signaling Technology Cat# 5174, RRID:AB_10622025), CD31 (Cell Signaling Technology Cat# 77699, RRID:AB_2722705) and Ki67 (Cell Signaling Technology Cat# 9449, RRID:AB_2797703) were purchased from Cell Signaling Technology (Danvers, MA, USA), and terminal deoxynucleotidyl transferase-mediated deoxyuridine triphosphate nick end-labeling (TUNEL) was purchased from Servicebio (Wuhan, Hubei, China). Sunitinib was purchased from Selleckchem (Houston, TX, USA) and anlotinib was provided by CHIATAI TIANQING Pharmaceutical Group (Nanjing, Jiangsu, China). Dimethyl sulfoxide and PEG400 (Sigma, Saint Louis, MO, USA) were used to dissolve sunitinib.

### 2.3. Cell Proliferation Assay and Clone Formation Assay

The inhibitory effects of anlotinib and sunitinib on cell proliferation were measured by Cell Counting Kit-8 (CCK-8, MedChemExpress, Monmouth Junction, NJ, USA) assay. SKOV-3 cells were cultured in 96-well plates at a density of 2000 cells per well overnight and then treated with anlotinib or sunitinib at the indicated concentrations. After incubated for 24, 48 or 72 h, 10 µL of CCK-8 working solution was added to the medium for 2 h at 37 °C. The resulting absorbance was measured at 450 nm by microplate reader (Thermo Fisher Scientific, Osterode am Harz, Niedersachsen, Germany). Prism version 8.4.0 (GraphPad Software, San Diego, CA, USA) was used to calculate the median inhibitory concentration (IC_50_). For the clone formation assay, SKOV-3 cells were seeded in 6-well plates at a density of 200 cells per well and cultured for 24 h. Then, the cells were treated with anlotinib or sunitinib for 12 days, a period in which visible colonies formed. After fixing with 4% paraformaldehyde for 10 min and staining by crystal violet for 15 min at room temperature, the colonies were visualized and quantified after extensive washed by Milli-Q water.

### 2.4. Wound-Healing Assay

SKOV-3 cells were seeded in 6-well plates and grown until they reached 90% confluence. The “wound” was subsequently created by a sterile 200 µL pipette tip, and then rinsed twice with FBS-free McCoy’s 5A medium to remove the nonadherent cells. Fresh medium containing anlotinib or sunitinib was subsequently added to the wells. After 48 h, images were obtained using a fluorescence microscope (Nikon, Tokyo, Japan). The distances of cell migration were measured by ImageJ software (Bethesda, MD, USA).

### 2.5. Cell Cycle Analysis and Cell Apoptosis

For the cell cycle assays, SKOV-3 cells were harvested after treatment with anlotinib at the indicated concentrations for 48 h. Single-cell suspension was fixed with 70% cold ethanol at 4 °C overnight. Samples were washed twice with cold PBS, and then the cell cycle distribution was analyzed using flow cytometer (FCM, ACEA Biosciences, San Diego, CA, USA) after staining by propidium iodide (PI)/RNase buffer (BD Biosciences, San Diego, CA, USA) at room temperature in the dark. For the apoptosis assays, SKOV-3 cells were incubated with anlotinib at the indicated concentrations for 24, 48 and 72 h. The cells were harvested and then detected using FCM after staining with annexin V-FITC and PI (BD Biosciences, San Diego, CA, USA) at room temperature in the dark. All assays were performed in triplicate.

### 2.6. Western Blot Analysis

After treatment with anlotinib at indicated concentrations for 48 h, SKOV-3 cells were washed with cold PBS twice and lysed with radio-immunoprecipitation assay lysis buffer containing protease and phosphatase inhibitor cocktails (MedChemExpress, Monmouth Junction, NJ, USA) on ice for 30 min. To remove insoluble materials, the cell lysate was centrifuged at 13,000× *g* for 15 min at 4 °C. The total protein concentration of the supernatants was measured by BCA protein assay kit (Thermo Fisher Scientific, Osterode am Harz, Niedersachsen, Germany). Protein was separated by 7.5% or 10% SDS-polyacrylamide gel electrophoresis and then transferred to polyvinylidene fluoride membranes (Merk Millipore, Darmstadt, Hessen, Germany). The membranes were reacted with specific primary antibodies overnight at 4 °C with gentle shaking after blocked by 5% nonfat milk for 1 h at room temperature. The immune complexes were reacted with HRP-conjugated secondary antibodies and made visible with chemiluminescence agents (Thermo Fisher Scientific, Osterode am Harz, Niedersachsen, Germany). The immunoblots were detected using the electrochemiluminescence imaging system (Bio-Rad, Berkeley, CA, USA).

### 2.7. Tumor Models of Peritoneal Metastases

Female BALB/c-nude mice (4–6 weeks old, body weight 18–20 g) were purchased from HFK Bioscience (Beijing, China) and housed under specific pathogen-free conditions in the State Key Laboratory of Biotherapy, Sichuan University. All animal experiments were approved by the Institutional Animal Care and Use Committee of Sichuan University (Chengdu, Sichuan, China) and performed in accordance with the guidelines of the Institutional Animal Care and Use Committee of Sichuan University (Chengdu, Sichuan, China). 5 × 10^6^ SKOV-3 cells suspended in 200 µL of McCoy’s 5A medium were intraperitoneally injected into female BALB/C-nude mice (day 0). After 7 days, all mice were randomly allocated into five groups (each group contained 5 mice) based on their body weights and received the indicated treatment. The mice were monitored daily for their living conditions, and weighed every 3 days. The mice were subsequently sacrificed after treatment by cervical dislocation method. Then, vital organs and tumor tissues were harvested, and the tumor weights were recorded.

### 2.8. Immunohistochemistry (IHC) and TUNEL Assay

Immunohistochemical analysis of Ki67 (1:500) and CD31 (1:2000) and TUNEL assays to detect the apoptosis rate were performed using paraffin sections of tumor tissues following the manufacturer’s instructions. All sections were observed and digitally photographed under a fluorescence microscope (Nikon, Tokyo, Japan). Five random fields were examined and counted for each section.

### 2.9. Hematoxylin-Eosin Staining

Tumor tissues and heart, liver, spleen, lung, and kidney paraffin sections were stained with hematoxylin-eosin (H&E) in accordance with the manufacturer’s instructions. All sections were observed and digitally photographed under microscope (Nikon, Tokyo, Japan).

### 2.10. Statistical Analysis

GraphPad Prism 8.4.0 software (GraphPad, San Diego, CA, USA) was used for all data analyses. All values are expressed as the mean ± standard deviation (SD). Significance was determined using unpaired t-test and one-way analysis of variance. We considered a value of *p* < 0.05 to be statistically significant.

### 2.11. Ethical Approval

All animal experiments were approved by the Institutional Animal Care and Use Committee of West China Hospital of Sichuan University (No. 20220104002) and performed in accordance with the guidelines of the Institutional Animal Care and Use Committee of West China Hospital of Sichuan University (Chengdu, Sichuan, China).

## 3. Results

### 3.1. Anlotinib Inhibited the Proliferation and Migration of SKOV-3 Cell In Vitro

**Figure 1:**
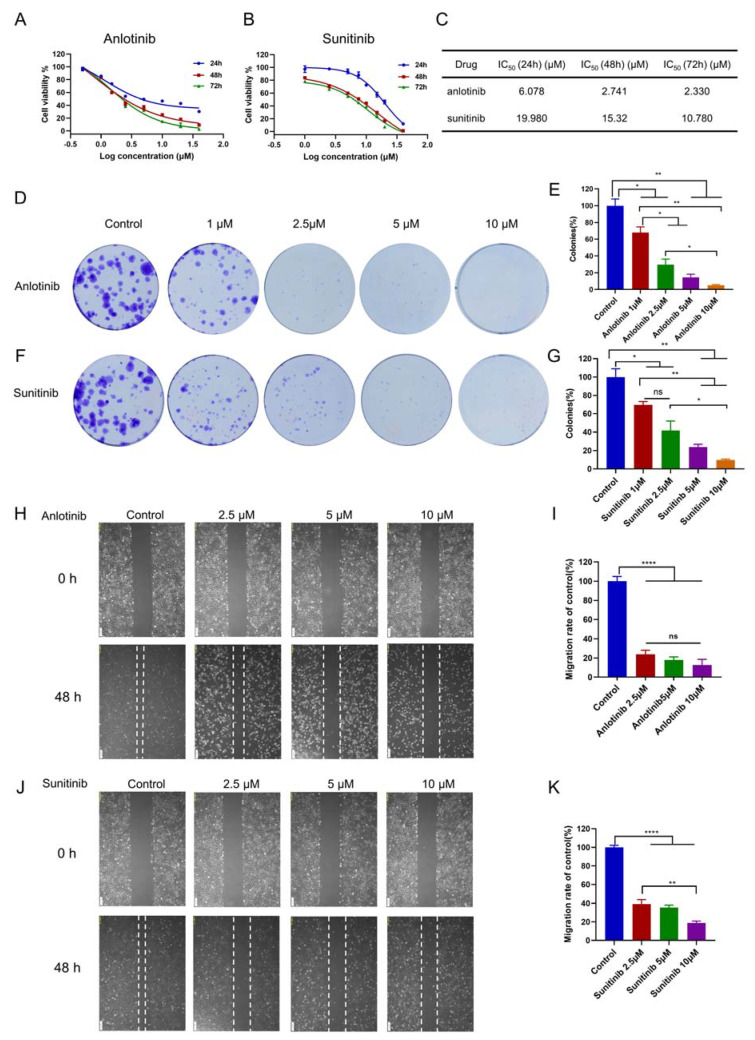
Anlotinib inhibited proliferation and migration of ovarian cancer cells. SKOV-3 cells were treated with anlotinib (**A**) or sunitinib (**B**) for 24, 48, or 72 h. CCK-8 assay was used to measure the viability of ovarian cancer cells, and IC_50_ values were calculated (**C**). (**D**,**F**) Representative images of colony formation assay. (**E**,**G**) The inhibition degrees of colony formation are shown in the histograms. Representative images of anlotinib (**H**) and sunitinib (**J**) inhibited SKOV-3 cells migration in wound-healing assay. (**I**,**K**) The degrees to which the wounds healed in the indicated groups are shown in the histograms. The data are presented as the mean ± SD from three independent experiments. * *p* < 0.05; ** *p* < 0.01; **** *p* < 0.0001; ns, non-significant.

To evaluate the effects of anlotinib and sunitinib on SKOV-3 cell growth, we incubated SKOV-3 cells with or without anlotinib and sunitinib at specific concentrations (0.5, 1, 1.5, 2.5, 5, 10, 20 or 40 µM) for 24, 48, and 72 h. We used the CCK-8 assay to assess cell viability and found that anlotinib and sunitinib both inhibited cell growth significantly in a dose- and time-dependent manner (Figure 1A,B). The IC_50_ values for the SKOV-3 cell line were presented in Figure 1C. The results suggested that the IC_50_ values of anlotinib were apparently lower than sunitinib. Moreover, we performed colony formation assays to detecte the anti-proliferative effects of anlotinib and sunitinib on ovarian cancer cells. The results revealed that anlotinib (Figure 1D,E) and sunitinib (Figure 1F,G) significantly inhibited clone numbers compared with the control group in SKOV-3 cell line. Furthermore, the wound-healing assays revealed that anlotinib effectively inhibited the migration of SKOV-3 cells (Figure 1H,I), and similar results were observed for sunitinib (Figure 1J,K). These results indicate that anlotinib and sunitinib both inhibited ovarian cancer cell proliferation and migration in vitro, and SKOV-3 cells were more sensitive to anlotinib.

### 3.2. Anlotinib Induced G2/M Arrest and Promoted Apoptosis of SKOV-3 Cell In Vitro

**Figure 2:**
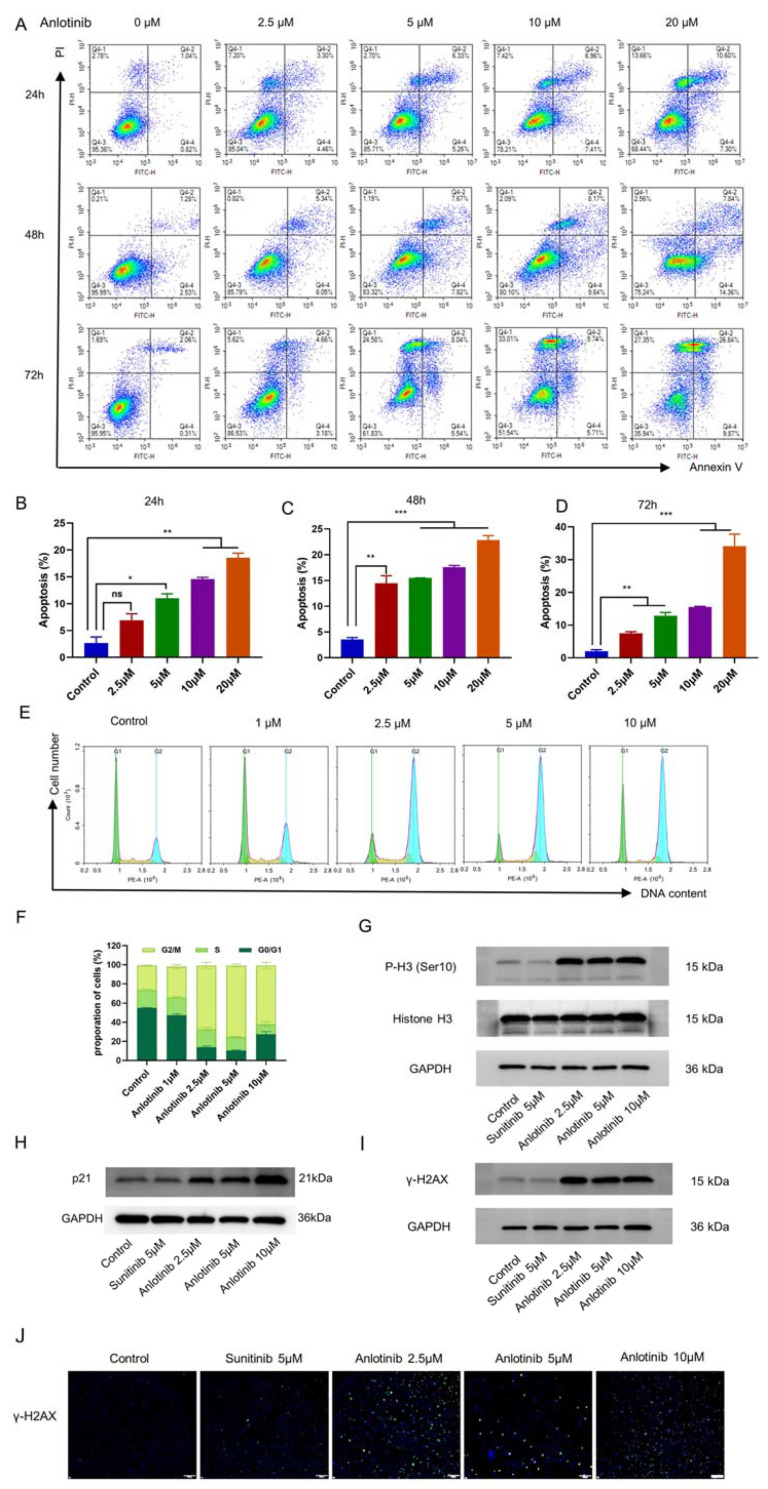
Anlotinib induced apoptosis and G2/M phase arrest of ovarian cancer cells. (**A**–**D**) The ratio of apoptotic cells was measured in SKOV-3 cells after anlotinib treatment for 24, 48, or 72 h. Apoptosis was detected using Annexin V-FITC and PI staining. The data are presented as the mean ± SD from three independent experiments. (**E**,**F**) Anlotinib induced G2/M phase arrest in SKOV-3 cells. The cells were treated with anlotinib at the indicated concentration for 48 h and analyzed by FCM after staining with PI. (**G**) The protein level of Histone H3 and its phosphorylation (P-H3 (Ser 10)) was detected by Western Blot in SKOV-3 cells treated with indicated concentration of anlotinib and sunitinib for 48 h. (**H**) The expression of p21 in SKOV-3 cells treated with indicated concentration of anlotinib and sunitinib for 48 h was detected by Western Blot. Phosphorylation of histone H2AX (γH2AX) was detected by western blot (**I**) and immunofluorescence staining (**J**). Bar = 200 μm; * *p* < 0.05; ** *p* < 0.01; *** *p* < 0.001; ns, non-significant.

To investigate the mechanism of the anti-proliferative effects of anlotinib in ovarian cancer cell, we evaluated apoptosis and cell cycle by FCM. The apoptosis rates of SKOV-3 cells were increased in a dose-dependent manner, but not in a time-dependent manner (Figure 2A–D). Moreover, to determine whether the inhibition of anlotinib on cell proliferation was the result of cell cycle arrest, we analyzed the cell cycle distributions of SKOV-3 cells by FCM with PI/RNase staining after treatment with anlotinib at the indicated concentrations (2.5, 5, 10 µM) for 48 h. As shown in Figure 2E,F, anlotinib significantly increased the percentage of SKOV-3 cells in G2/M phase and decreased the number of cells in G0/G1 phase in a dose-dependent manner. Increase in the phosphorylation of histone H3 (Ser 10) further indicated G2/M arrest (Figure 2G). The expression level of p21 protein in SKOV-3 cells was detected by western blot after treatment with the indicated concentrations of anlotinib and sunitinib for 48 h (Figure 2H). The results showed that anlotinib significantly upregulated the expression of p21 protein in vitro. Moreover, we found that anlotinib could induce DNA damage and increase γH2AX at 48 h after dosing in SKOV3 cell line (Figure 2I,J). Collectively, these results suggest that anlotinib induced G2/M phase arrest, apoptosis and DNA damage of ovarian cancer cell in vitro, which contributed to its growth inhibition.

### 3.3. Anlotinib Effectively Inhibited Tumor Growth in Ovarian Carcinoma Peritoneal Metastasis Model

**Figure 3:**
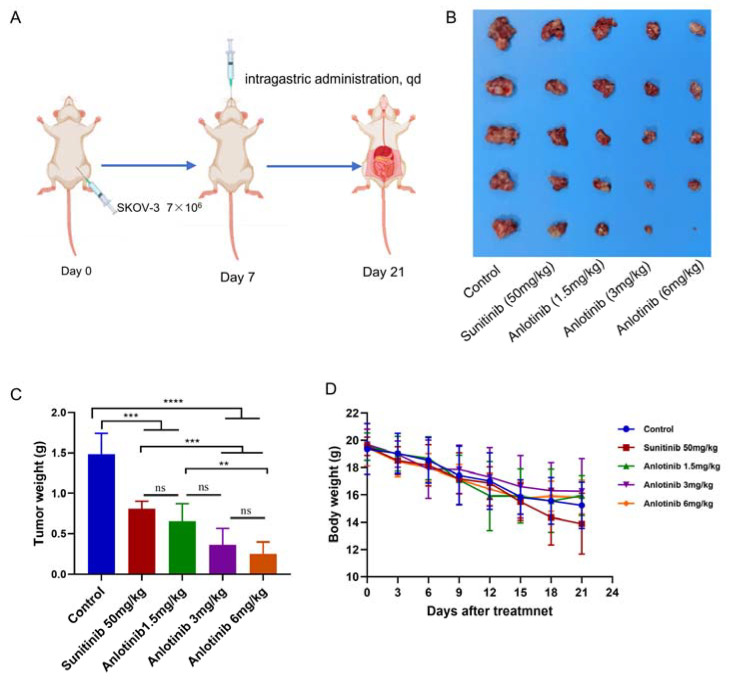
Anlotinib exhibited effective antitumor activity in vivo. (**A**) Timeline of the experiments in vivo. (**B**) SKOV-3 cells were intraperitoneally injected into female BALB/C-nude mice, which were treated with an oral dose of 1.5, 3 or 6 mg/kg anlotinib, 50 mg/kg sunitinib or vehicle once daily for 2 weeks. The image shows the sizes of tumors at the end of experiment. (**C**) The weights of tumors were measured. The data are presented as the mean ± SD. ** *p* < 0.01; *** *p* < 0.001; **** *p* < 0.0001; ns, non-significant. (**D**) Mice weights were calculated every 3 days.

Anlotinib has demonstrated potent antitumor activity in several solid tumors [21]. However, its effect on ovarian cancer has never been reported. To evaluate the antitumor potential of anlotinib in vivo, we established ovarian cancer peritoneal metastasis tumor model. Briefly, SKOV-3 cells were intraperitoneally injected into female BALB/C-nude mice, which received an oral dose of 1.5, 3 or 6 mg/kg anlotinib, 50 mg/kg sunitinib or vehicle once daily for 2 weeks after inoculated 7 days (Figure 3A). As shown in Figure 3B,C, anlotinib and sunitinib significantly inhibited ovarian tumor growth compared to the vehicle-treated group. In addition, the mouse body weight of the sunitinib-treated group continued to decrease (Figure 3D).

### 3.4. Anlotinib Inhibited Tumor Cells Proliferation and Angiogenesis with Low Drug Toxicity In Vivo

**Figure 4:**
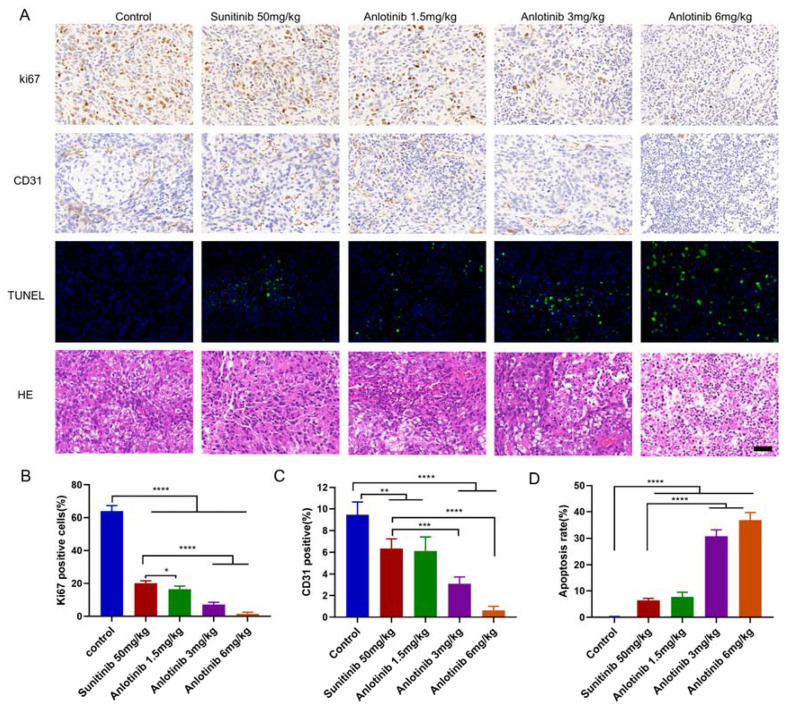
Anlotinib effectively inhibited tumor growth and angiogenesis, induced apoptosis in ovarian carcinoma peritoneal metastases model. (**A**) Representative images of TUNEL and IHC staining of Ki67 and CD31, and H&E staining of tumor tissues from various groups. Bar = 100 μm (**B**) Ki67 positive rate of tumor tissues from various groups. (**C**) CD31 positive rate of tumor tissues from various groups. (**D**) Apoptosis rate of tumor tissues from various groups. The data are presented as the mean ± SD from three independent experiments. * *p* < 0.05; ** *p* < 0.01; *** *p* < 0.001; **** *p* < 0.0001.

**Figure 5:**
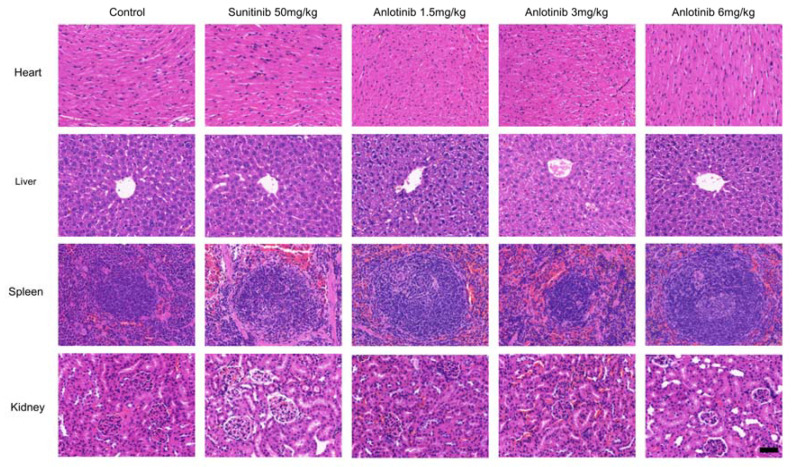
Representative images of H&E staining of heart, liver, spleen and kidney from the control and treatment groups. Bar = 100 μm.

To investigate the mechanism by which anlotinib suppresses tumor growth in vivo, biomarkers for tumor cell proliferation, apoptosis and angiogenesis were also detected (Figure 4A). The results showed that anlotinib and sunitinib markedly inhibited cell proliferation (the percentage of Ki67-positive cells) and the apoptotic rates of anlotinib-treated groups were markedly increased (Figure 4B,D). Furthermore, anlotinib and sunitinib markedly inhibited angiogenesis (the percentage of CD31-positive cells) (Figure 4C). In addition, we noted no abnormal organ morphology after treatment with anlotinib and sunitinib compared to vehicle-treated (Figure 5). Taken together, these data suggested that anlotinib suppressed tumor growth by inhibiting cell proliferation and angiogenesis in peritoneal metastasis tumor model.

## 4. Discussion

Targeted therapy is one of the major modalities of medical treatment for ovarian cancer [24]. Anlotinib has shown broad antitumor effects as a broad-spectrum anti-angiogenic agent [11,13,25,26]. However, some studies demonstrated that anlotinib directly inhibited tumor cells rather than angiogenesis [15,22,27,28,29], and an increasing number of unconventional pharmacologic targets have been identified [15,22,30,31], such as EGFR and c-Myc. Recent studies have shown that anlotinib induced mitochondrial dysfunction related to the impairment of complexes IV and V and directly induced apoptosis by ROS in gastric cancer cells [21]. Moreover, Liang et al. found that anlotinib directly targeted EGFR via a dual mechanism by simultaneous inhibitory effects on cancer and endothelial cells [22]. A retrospective study confirmed that anlotinib was promising efficacy and well tolerated in platinum-resistant ovarian cancer [32], but their data are limited representative due to the lower number of cases. Although several clinical studies are ongoing, the response of ovarian cancer cells to anlotinib remains poorly understood.

In this study, we found that anlotinib directly inhibited ovarian cell proliferation by inducing cell apoptosis and G2/M phase arrest both in vivo and in vitro. The p21 protein as a cyclin-dependent kinase inhibitor participates in a broader range of biological functions, including regulation of transcription, DNA repair, cell motility, apoptosis and cell differentiation [33,34,35]. Song et al. reported that anlotinib significantly upregulated the expression of p21 in HCCC9810 and REB cells of intrahepatic cholangiocarcinoma [18]. Consistent with the previous study, we also found that anlotinib upregulated p21 in SKOV-3 cells, while sunitinib did not (Figure 2C). Additionally, anlotinib exerted potent anti-angiogenic effects without blocking VEGFR2 in ovarian cancer models. The clear mechanism of anlotinib against angiogenesis in ovarian cancer needs further exploration.

In the present study, we found that the antitumor activity of anlotinib at 1.5 mg/kg was comparable to that of the well-known sunitinib at 50 mg/kg (Figure 3B). Moreover, the tumor weights of the anlotinib 3 mg/kg group and 6 mg/kg group were significantly lower than of the sunitinib 50 mg/kg group (*p* < 0.001). Further potential mechanisms study also showed that the effective dose of anlotinib at 1.5 mg/kg daily was significantly lower than sunitinib (Figure 4A), which require 40–50 mg/kg to achieve significant inhibition of tumor growth in mice [36].

## 5. Conclusions

Anlotinib inhibited ovarian cancer cell proliferation by inducing G2/M phase arrest and promoting apoptosis both in vivo and in vitro. Anlotinib was more effective than sunitinib in anti-ovarian cancer. The results of our preclinical study can serve as a proof of concept for anlotinib treatment of ovarian cancer.

## Data Availability

Not applicable.
